# Motion as Medicine: Physical Activity, Joint Sensitivity, and Pain Management—A Narrative Review

**DOI:** 10.3390/medsci14040495

**Published:** 2026-08-19

**Authors:** Luminita Labusca, Bogdan Puha, Bianca-Ana Dmour, Ilie Onu, Mihaela Camelia Tirnovanu, Ștefan-Dragoș Tîrnovanu, Awad Dmour

**Affiliations:** 1 National Institute of Research and Development for Technical Physics, 700050 Iasi, Romania; llabusca@phys-iasi.ro; 2 Department of Orthopedics and Trauma/Surgical Science (II), Faculty of Medicine, Grigore T. Popa University of Medicine and Pharmacy Iasi, 16 University Street, 700020 Iasi, Romania; bianca-ana.dmour@umfiasi.ro (B.-A.D.); stefan-dragos.tirnovanu@umfiasi.ro (Ș.-D.T.); dmour-awad@umfiasi.ro (A.D.); 3 Grigore T. Popa University of Medicine and Pharmacy, 700115 Iasi, Romania; ilie.onu@umfiasi.ro (I.O.); mihaela.tirnovanu@umfiasi.ro (M.C.T.); 4 Cardiology Clinic, “St. Spiridon” County Clinical Emergency Hospital, 700111 Iasi, Romania

**Keywords:** physical activity, synovial joint, joint homeostasis, mechanotransduction, osteoarthritis, pain modulation, adaptive loading window

## Abstract

Background: Physical activity is widely recommended for preserving musculoskeletal health and managing osteoarthritis-related pain, although its benefits are commonly framed in terms of muscle strengthening, weight control, and physical performance. This narrative review aimed to examine movement more broadly as a physiological regulator of synovial joint homeostasis, sensory calibration, and functional adaptation. Methods: A structured literature search was performed in PubMed/MEDLINE, Scopus, and Web of Science from database inception to 1 February 2026. Experimental studies, observational studies, clinical trials, systematic reviews, meta-analyses, and selected narrative reviews addressing movement-responsive joint biology or pain regulation were considered. Evidence was synthesized across four interrelated domains: mechanical, fluidic, immune-metabolic, and sensory regulation. Results: The narrative synthesis indicates that the concept of the synovial joint as a dynamic mechano-fluidic organ in which cartilage, synovium, synovial fluid, capsule, subchondral bone, periarticular tissues, and sensory pathways interact continuously. Repeated physiological movement may promote synovial fluid exchange, lubrication, cartilage nutrition, hyaluronic acid and lubricin function, matrix turnover, anti-inflammatory signaling, proprioceptive control, and exercise-induced hypoalgesia. In contrast, inactivity and unloading may impair fluid dynamics, promote muscle inhibition, stiffness, inflammatory persistence, sensory deconditioning, and loss of function. Excessive or poorly distributed loading may also disrupt homeostasis through matrix injury, inflammation, fatigue, and nociceptive sensitization. These findings informed the proposed adaptive loading window, a hypothesis-generating conceptual framework rather than a clinically validated threshold, describing the dynamic range of movement within which joint function and pain regulation may be supported without sustained tissue or symptom aggravation. Conclusions: Movement should be viewed not only as a therapeutic intervention, but also as a continuous regulator of joint biology and perception. Its clinical value may depend on identifying an individualized loading range that supports adaptation, function, and confidence in movement while avoiding both underloading and overload.

## 1. Introduction

Movement is a fundamental component of musculoskeletal development and function. In mammals, the musculoskeletal system provides the structural basis for body motion [1], but its function depends on coordinated vascular, lymphatic, neural, metabolic, and immune support. Mechanical forces generated by embryonic and fetal movements contribute to joint morphogenesis, cavitation, and tissue differentiation [2], while reduced movement during critical developmental periods has been associated with impaired joint formation and altered musculoskeletal architecture [3,4]. These observations indicate that movement is not merely an output of the musculoskeletal system, but also a biological stimulus capable of shaping joint development and maintaining tissue responsiveness.

If function contributes to the preservation of organ and system integrity [4] and supports tissue and whole-body homeostasis [5], movement may be understood not only as a manifestation of musculoskeletal health, but also as an active contributor to the maintenance and adaptability of the biological systems that sustain joint function across the lifespan.

Physical activity is commonly framed either as a component of competitive or recreational sport or as an intervention used to prevent or treat disease in aging or injured joints. We propose a broader physiological perspective in which movement is not merely prescribed to preserve or rehabilitate synovial joints, but represents one of the conditions through which they develop, adapt, and remain functional.

Contemporary evidence supports exercise as a core intervention for osteoarthritis and other musculoskeletal conditions, with demonstrated benefits for pain, physical function, strength, and quality of life. However, considerable heterogeneity persists regarding exercise modality, intensity, duration, and individual response, and a universally applicable dose-response relationship has not been established. Recent research has also extended beyond conventional functional outcomes to examine inflammatory, sensory, and pain-modulatory responses to exercise. Nevertheless, these effects are often investigated within separate clinical or biological domains, while the relationships among mechanical loading, synovial fluid dynamics, tissue mechanobiology, immune regulation, and joint sensory processing remain incompletely integrated within a unified joint-organ framework.

For clarity, several related terms used throughout this review are distinguished according to context. Movement refers broadly to displacement of the body or a joint, whereas physical activity refers to bodily movement generated by skeletal muscle that requires energy expenditure. Exercise represents a planned, structured, and repetitive form of physical activity undertaken to maintain or improve physical function or fitness. Mechanical loading refers specifically to the forces, stresses, and strains transmitted to joint tissues during movement, weight bearing, or externally applied stimulation. Motion, retained in the title as a broader conceptual term, refers to movement as a general physiological phenomenon. These concepts are closely related but are not considered interchangeable throughout this review.

Against this background, the present review examines movement as a continuous regulator of joint biology [6]. and perception, integrating developmental, mechanical, fluidic, immune-metabolic, and sensory evidence relevant to synovial homeostasis, structural adaptation, and pain regulation.

**Figure 1:**
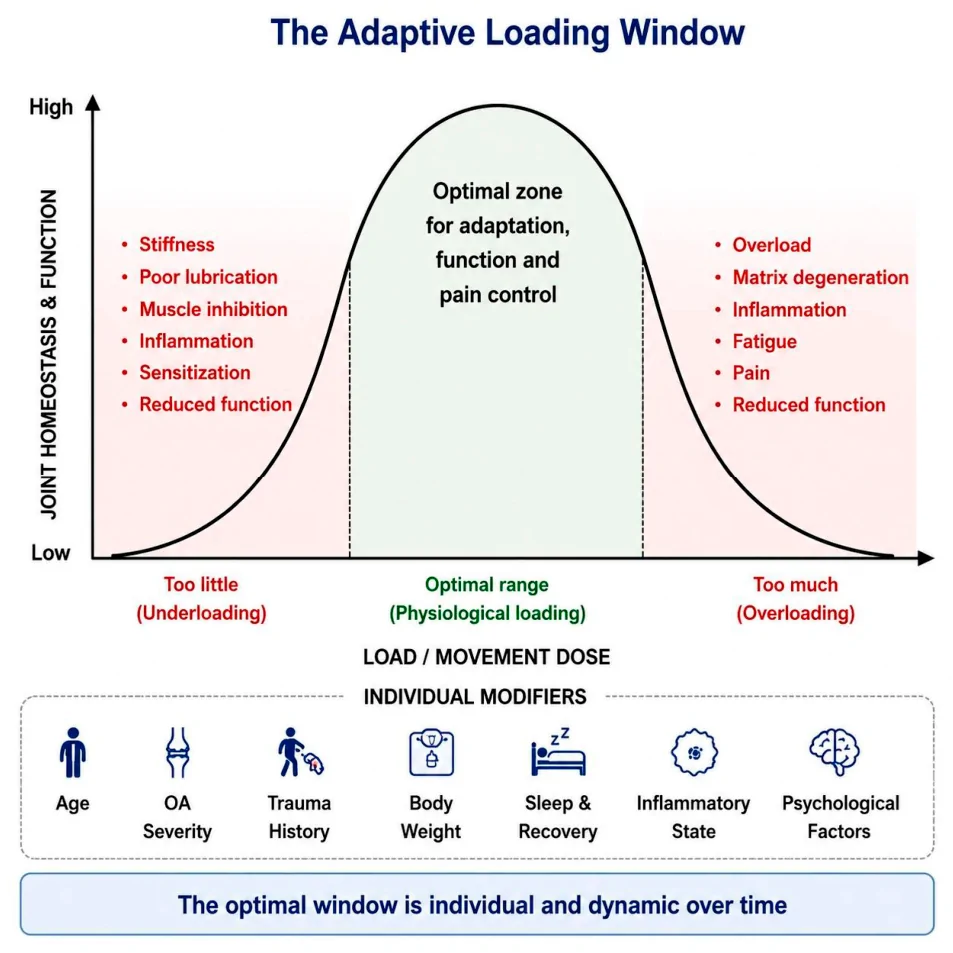
Adaptive Loading Window.

The adaptive loading window overlaps conceptually with established principles of graded exercise, optimal loading, and load management, but differs in its intended level of integration. Rather than defining an exercise dose primarily according to symptoms, performance, or tissue tolerance, the present framework considers mechanical exposure together with fluidic, inflammatory, metabolic, and sensory responses of the joint organ. It should therefore be regarded as a hypothesis-generating extension of existing loading concepts rather than a validated alternative to them. Within this framework, both insufficient and excessive mechanical stimulation may become maladaptive, while an individualized intermediate range may support joint homeostasis, functional adaptation, and pain regulation (Figure 1).

Within this framework, pain is considered not simply an outcome of structural pathology, but part of a movement-responsive sensory system influenced by mechanical, inflammatory, peripheral nociceptive, and central regulatory processes. This perspective may broaden the role of movement in pain management beyond improvements in strength and mobility to include modulation of nociceptive sensitivity, proprioceptive control, exercise-induced hypoalgesia, and confidence in movement.

Accordingly, this narrative review has a conceptual and hypothesis-generating objective. It integrates developmental, mechanical, fluidic, immune-metabolic, and sensory observations to explore the proposition that movement and mechanical loading may act as interconnected regulators of synovial joint homeostasis and pain-related sensory processes. On this basis, we propose the adaptive loading framework as an authors’ conceptual synthesis intended to organize existing observations and generate testable hypotheses across development, injury, osteoarthritis, and aging. The proposed approach is not intended to represent an evidence-graded or clinically validated model; rather, the proposed relationships outlined here provide elements for further investigation that may be examined through appropriately designed mechanistic, longitudinal, imaging, biomarker, and interventional studies.

## 2. Materials and Methods

This article was designed as a narrative, concept-generating review examining movement as a regulator of synovial joint biology and pain. A structured literature search was conducted in PubMed/MEDLINE, Scopus, and Web of Science from database inception to 1 February 2026. The search combined terms related to movement and mechanical loading with terms describing joint homeostasis, mechanobiology, synovial fluid, cartilage, synovium, inflammation, nociception, and rehabilitation.

A representative PubMed search strategy was: (“movement” OR “physical activity” OR “exercise” OR “mechanical loading” OR “immobilization”) AND (“synovial joint” OR “joint homeostasis” OR “mechanotransduction” OR “synovial fluid” OR “hyaluronic acid” OR “lubricin” OR “PRG4” OR “cartilage” OR “synovium” OR “synovial macrophages” OR “infrapatellar fat pad”) AND (“pain” OR “nociception” OR “proprioception” OR “exercise-induced hypoalgesia” OR “osteoarthritis” OR “joint trauma” OR “rehabilitation” OR “healthy aging”). Database-specific syntax and controlled vocabulary, including MeSH terms where applicable, were adapted for each database. Reference lists of relevant articles were also examined to identify additional publications.

Eligible publications included original experimental studies, human observational studies, clinical trials, systematic reviews, meta-analyses, and selected authoritative narrative reviews addressing movement-responsive joint biology, mechanical loading, pain regulation, or rehabilitation. Animal and in vitro studies were included when they provided mechanistic information not readily obtainable from human studies. Studies focused exclusively on athletic performance without a clear relationship to joint biology, pain, rehabilitation, or functional adaptation were not prioritized. The review focused on full-text articles published in English. Conference abstracts, editorials, letters, and publications without sufficient methodological or mechanistic information to inform the review framework were not included as primary evidence. Duplicate publications identified across database searches were considered only once.

Potentially relevant publications were assessed according to their relevance to the biological and clinical questions addressed by the review. Priority was given to recent systematic reviews, meta-analyses, clinical trials, human mechanistic studies, and influential experimental investigations. Older studies were retained when they provided foundational evidence concerning joint development, synovial fluid physiology, mechanotransduction, or the consequences of immobilization. Where multiple publications addressed similar questions, preference was given to more recent or methodologically stronger evidence while retaining seminal studies when historically or mechanistically relevant.

Evidence was synthesized thematically across four interacting domains: mechanical, fluidic, immune-metabolic, and sensory regulation. These domains emerged iteratively during literature synthesis from recurring mechanisms linking movement with joint development, tissue homeostasis, inflammatory regulation, and sensory processing. The adaptive loading window was subsequently developed as an integrative conceptual framework describing the dynamic range within which mechanical stimulation may support joint homeostasis, functional adaptation, and pain regulation without sustained tissue injury or symptom aggravation. Evidence derived from human clinical studies was distinguished from findings obtained in animal models, tissue explants, or cell-culture experiments, and mechanistic extrapolations not directly demonstrated in humans were interpreted cautiously.

As this was a narrative and hypothesis-generating review, no formal risk-of-bias assessment, quantitative meta-analysis, or certainty-of-evidence grading was undertaken. The methodological and reporting quality of the review was evaluated with reference to the Scale for the Assessment of Narrative Review Articles (SANRA) [7], including justification of the review, clarity of aims, transparency of the literature search, appropriate referencing, scientific reasoning, and presentation of relevant evidence.

Evidence synthesis and interpretation are presented together within thematic sections, consistent with the narrative-review design; statements representing the authors’ conceptual interpretation, particularly the adaptive loading window, are explicitly distinguished from findings directly supported by the reviewed literature.

## 3. Revisiting Synovial Joint Developmental Anatomy and Function

During development, movement is not merely an emerging function of synovial joints but also an important biological influence on their formation. Embryonic and fetal movements contribute to joint morphogenesis and cavitation. Experimental reduction of muscle-driven movement in developing animal models has been associated with cavitation defects, altered joint morphology, and impaired postnatal function. In developing mouse limbs, synovial cavity formation begins through microcavities within the interzone and is associated with skeletal flexion, elongation, and hyaluronic acid accumulation [8]. In humans, prolonged absence of fetal movement (akinesia) is associated with congenital joint stiffness, as observed in conditions such as arthrogryposis [9], supporting the role of mechanical stimuli as formative biological signals rather than merely functional outputs.

Synovial joint development begins early in embryogenesis, with a relatively homogeneous interzone appearing at future joint sites in humans around the sixth embryonic week and a three-layered interzone becoming evident by approximately the seventh week. In larger joints, cavitation and organization of the joint cavity continue during subsequent fetal development. The interzone contains a specialized population of joint-forming progenitor cells, classically associated with GDF5 expression, that subsequently contribute to intra-articular joint tissues, including articular cartilage, synovial lining, ligaments, joint capsule, knee menisci, and the intra-articular extrasynovial fat pad [10]. In mouse embryonic limbs, synovial cavity formation has been shown to begin around E15.0, and recent evidence supports cavitation as an active mechano-osmoregulatory process involving ion pumps, aquaporin-1, mechanosensing genes, and lubricant gene expression [11,12].

At the cellular level, developing joint tissues exhibit early mechanosensitivity, reflecting an intrinsic capacity to detect and respond to physical cues. Interzone cells and adjacent chondrogenic populations express components of canonical mechanotransduction machinery, including primary cilia, integrin-mediated adhesion complexes, stretch-activated ion channels such as TRPV4 and Piezo family members, and cytoskeleton-associated signaling pathways [13,14,15]. These structures enable the conversion of mechanical stimuli generated by embryonic movement into intracellular signals that regulate gene expression, cell fate decisions, and extracellular matrix organization. Mechanical cues have been shown in vitro and in animal models to influence chondrogenic differentiation, joint patterning, and the spatial organization of developing tissues. Calcium signaling, MAPK cascades, and transcriptional regulators such as YAP/TAZ are involved in mechanosensing and contribute to both joint homeostasis and disease [16,17]. In this context, movement does not merely modulate an already formed structure, but interacts with a developmentally primed cellular system capable of translating physical forces into morphogenetic responses.

During joint development, the synovium is also colonized by monocyte-derived populations that contribute to the establishment of tissue-resident macrophages and lifelong immune surveillance [18]. In mice, embryonic synovial macrophages derived from extraembryonic yolk sac progenitors are already present in the prenatal joint, while bone marrow-derived hematopoietic synovial macrophages appear around the perinatal period and subsequently coexist with embryo-derived populations [19]. In humans, tissue-resident macrophage ontogeny is similarly considered to involve sequential yolk sac and fetal liver hematopoietic waves, later complemented by postnatal bone marrow-derived monocyte influx. Resident synovial macrophages are maintained locally and, in adulthood, include lining macrophages that form a CX3CR1-positive protective barrier separating the synovial cavity from the sublining tissue. This places immune cells at an anatomical interface continuously exposed to motion-dependent fluid shifts, pressure changes, and matrix deformation [20]. Although macrophages are known experimentally to respond to mechanical properties of their environment, including substrate stiffness, confinement, and cytoskeletal deformation [21], whether developmental joint movement directly influences synovial macrophage positioning or maturation of this barrier remains insufficiently defined to date.

**Figure 2:**
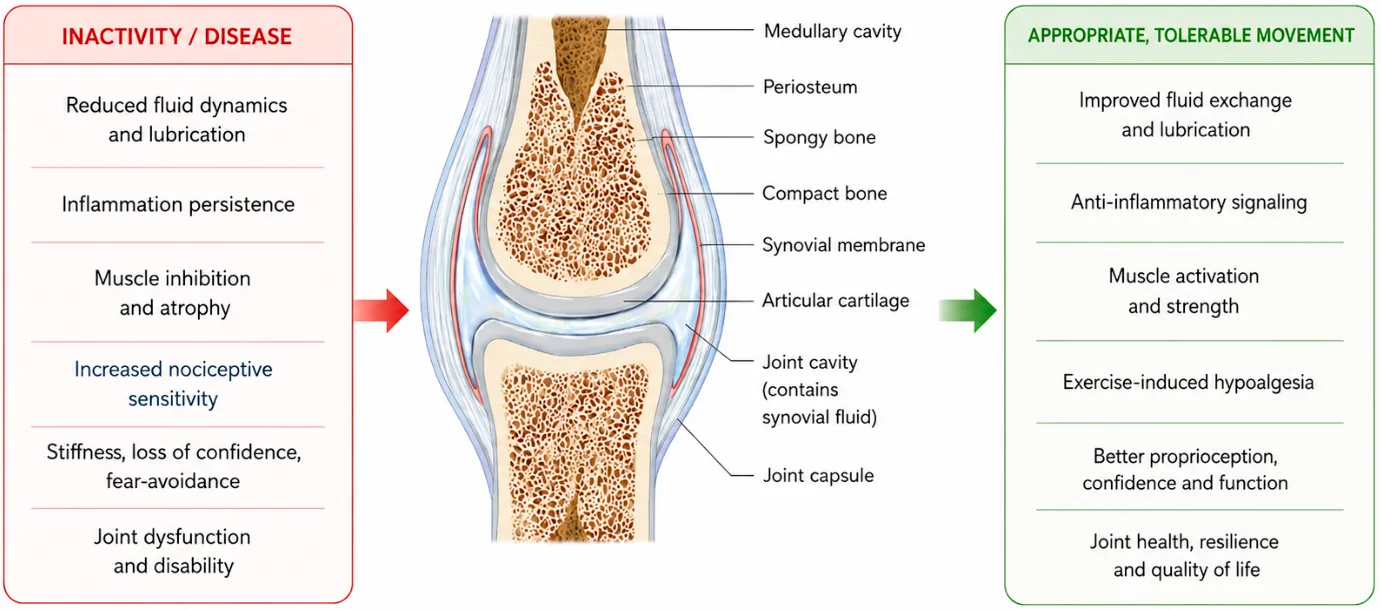
The balance of movement and loading. Deficient movement may impair joint morphogenesis, lubrication, matrix maintenance, sensory conditioning, and functional recovery. Physiological movement supports lubrication, matrix turnover, immune balance, sensory calibration, and joint homeostasis, whereas excessive or poorly distributed loading may promote altered joint shaping, matrix degradation, inflammation, fatigue, and loss of adaptive capacity.

More broadly, the available developmental evidence indicates that the biological response to mechanical stimulation is unlikely to follow a simple linear relationship. Both insufficient movement and excessive or abnormal loading may interfere with joint morphogenesis and tissue organization, whereas physiological movement within an appropriate range appears to support normal development and maturation. This developmental pattern provides an early biological basis for the broader concept of an adaptive loading window explored throughout this review and illustrated in Figure 2.

## 4. The Synovial Joint as a Mechano-Fluidic System

**Figure 3:**
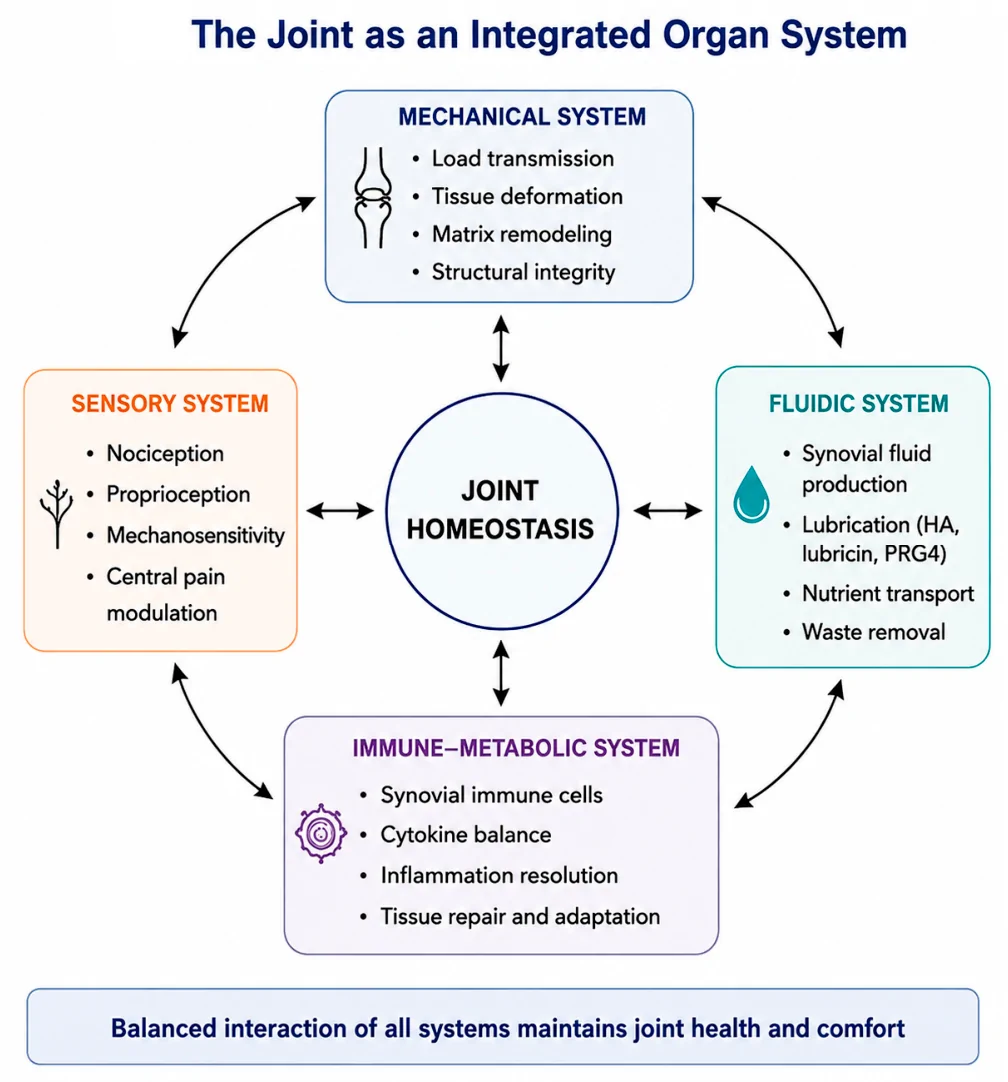
The joint as an integrated organ.

Synovial joints function not only as load-bearing mechanical structures but as dynamic mechano-fluidic systems in which movement governs the distribution, composition, and functional properties of synovial fluid [22,23] (Figure 3). Cyclical joint loading and unloading contribute to synovial fluid movement, convective transport, lubrication, and metabolic exchange. During motion, variations in intra-articular pressure and cartilage deformation promote fluid displacement across the joint cavity and through the porous cartilage matrix, supporting nutrient delivery to avascular tissues and clearance of metabolic by-products.

Because articular cartilage is avascular, chondrocyte metabolism depends largely on solute transport from synovial fluid and subchondral sources. Cyclic loading has long been investigated as a mechanism that may facilitate fluid movement and solute transport through cartilage, although diffusion remains an important contributor depending on molecule size, matrix permeability, and loading conditions [24].

Movement contributes to the distribution of synovial fluid across the joint space, supporting low-friction articulation and maintaining contact between cartilage surfaces and lubricant molecules. Synovial fluid lubrication depends on the coordinated activity of hyaluronan (HA), lubricin/PRG4, and surface-active phospholipids, with HA and PRG4 acting synergistically in boundary and mixed lubrication regimes [25].

Synovial fluid composition is closely linked to the activity and phenotype of joint-resident cells. Fibroblast-like synoviocytes (FLS) and superficial-zone chondrocytes contribute to HA and lubricin/PRG4 production. FLS can respond directly to mechanical stimulation, with mechanosensitive HA secretion demonstrated in vitro [26,27]. PRG4 expression in superficial zone chondrocytes has been shown to be modulated by mechanical loading in cartilage explant systems and in rodent models [28,29]. However, direct evidence demonstrating coordinated, short-term modulation of synovial fluid (SF) composition in response to movement in humans remains limited, and most available data derive from in vitro or tissue-specific models.

SF cannot be regarded as a simple Newtonian lubricant. SF displays viscoelastic, shear-thinning, and thixotropic properties: at low shear or relative rest it behaves as a more viscous protective medium, while during movement and higher shear it becomes less viscous, allowing easier sliding between articular surfaces [30].

Beyond its classical description as a homogeneous viscoelastic lubricant, synovial fluid is increasingly recognized as a structurally complex, multiscale system. Recent experimental observations suggest that PRG4 can form discrete gel-like aggregates whose size and mechanical behavior are influenced by the surrounding HA matrix, supporting a hierarchical organization of synovial fluid rather than a simple homogeneous continuum [31].

These observations suggest that lubrication depends on dynamic interactions among fluid viscosity, boundary-layer organization, and load-dependent redistribution of synovial fluid constituents. Mechanical loading may therefore influence both the bulk rheological behavior and microstructural organization of synovial fluid. Movement, by imposing shear, compression, and fluid displacement, is therefore likely to influence not only the bulk rheological properties of synovial fluid but also its microstructural organization.

Other intra-articular structures, ligaments and menisci, may also participate in shaping the biochemical and mechanical environment of the joint, although their contribution to synovial fluid remodeling remains less well characterized.

Collectively, these findings support a reciprocal relationship between mechanical loading and the synovial fluid environment. Movement alters pressure, shear, fluid displacement, and tissue deformation, while synovial fluid properties influence lubrication, cartilage nutrition, and the mechanical conditions experienced by joint-resident cells. However, much of the mechanistic evidence derives from in vitro, explant, or animal models, and the type, frequency, and magnitude of movement required to optimize these processes in humans remain insufficiently defined. The synovial joint is therefore best considered as an integrated mechano-fluidic system in which mechanical and fluidic processes are interdependent.

## 5. Synovial Joint Homeostasis Under Mechanical Loading

Synovial joint homeostasis depends on continuous crosstalk among mechanical forces, synovial lining cells, chondrocytes, cellular elements of the tidemark and subchondral bone, and the molecular composition of synovial fluid. Under physiological conditions, movement generates recurrent mechanical stimuli through loading and unloading, shear stress, variations in intra-articular pressure, and fluid displacement. These forces are not merely external physical events; they are detected by joint-resident cells and translated into biochemical responses that contribute to the production, turnover, and maintenance of synovial fluid.

Fibroblast-like synoviocytes (FLS) and superficial-zone chondrocytes are important mechanosensitive contributors to synovial fluid homeostasis. Experimental studies demonstrate that mechanical stimulation modifies HA secretion and pathways involved in HA, PRG4/lubricin, and hyaluronidase regulation [32,33], while both fluid-flow shear and in vivo running can increase Prg4 expression through defined mechanotransduction pathways [34]. These findings support load-dependent regulation of lubricant-producing cellular phenotypes, although the relative contribution of different cell populations varies according to tissue, loading regimen, developmental stage, and inflammatory context.

Importantly, these responses do not appear to increase linearly with mechanical stimulation. Immobilization and unloading are associated with reduced synovial fluid HA and altered synovial cell activity [35,36], whereas experimental unloading can produce early synovitis and fibrosis involving activated fibroblast and macrophage populations [36]. Together, these findings support a loading-dependent regulation of synovial homeostasis while emphasizing that both insufficient and excessive mechanical exposure may become maladaptive.

Articular cartilage also contributes actively to synovial joint homeostasis and to the quantity and composition of synovial fluid. Superficial-zone chondrocytes are highly mechanosensitive and participate in maintaining the boundary-lubricating interface through the expression of PRG4/lubricin [37]. In vitro, dynamic shear stimulation of bovine cartilage increased PRG4 biosynthesis, whereas static and dynamic compression were also shown to regulate PRG4 metabolism, indicating that lubricant production by cartilage is sensitive to both the type and magnitude of mechanical input [38]. Studies of articular surface motion have further demonstrated that chondrocyte subpopulations respond differently to mechanical stimulation, supporting the interpretation of the superficial zone as a specialized movement-responsive compartment [39]. In mice, voluntary wheel running increased Prg4 expression in the superficial zone of knee articular cartilage through COX-2-dependent and CREB-related signaling mechanisms [28].

Clinical studies have identified alterations in PRG4/lubricin and HA concentrations in the synovial fluid of patients with osteoarthritis or following joint injury, together with diminished cartilage-lubricating capacity of osteoarthritic synovial fluid [40]. Overall, cartilage mechanosensitivity is well supported experimentally, but translation to humans remains incomplete, particularly regarding the dose and type of physical activity required to modify PRG4 production or synovial fluid composition clinically. However, direct clinical evidence demonstrating that a clearly defined exercise or loading intervention acutely modifies cartilage-derived PRG4 production remains limited. In particular, clinical data relating the dose, intensity, duration, or magnitude of physical activity to the amount and composition of synovial fluid are still lacking.

The tidemark, calcified cartilage, and subchondral bone form an osteochondral interface through which movement-derived forces are transmitted and dissipated, while also acting directly upon local cellular populations. Morphometric studies of human postmortem specimens have shown that calcified-zone thickness decreases with age, whereas tidemark remodeling increases in regions corresponding to pressure points, suggesting an age-related adaptation of the joint to mechanical loading [41]. Within subchondral bone, movement-derived mechanical stimuli are continuously interpreted by osteocytes, osteoblasts, and osteoclasts through mechanotransduction pathways involving calcium signaling, Piezo1, and YAP-dependent transcriptional regulation. These pathways coordinate bone remodeling, cartilage support, and structural adaptation to loading [42,43,44].

In vivo studies further demonstrate that subchondral bone is rapidly remodeled in response to altered mechanical loading. Increased or abnormal cyclic loading can induce subchondral bone thickening and trabecular changes, whereas unloading or immobilization promotes subchondral bone atrophy, marrow expansion, increased osteoclastic activity at the osteochondral junction, and remodeling of the tidemark and calcified cartilage [45]. In a non-invasive mouse knee-loading model, intermittent mechanical loading induced subchondral bone thickening and intensified lesions in the overlying cartilage. The subchondral compartment should therefore be regarded as a movement-responsive load-distribution interface that contributes to coordinated mechanosensitive responses throughout the joint [46].

Ligaments contribute to synovial joint homeostasis by maintaining stability, guiding physiological motion, restricting abnormal displacement, and preserving the mechanical conditions under which cartilage, synovium, menisci, and subchondral bone function. Ligament fibroblasts are themselves mechanosensitive. Mechanical stretch regulates collagen expression, matrix-remodeling enzymes, and growth-factor signaling, whereas immobilization or injurious strain impairs matrix organization and mechanical competence. Ligaments therefore contribute to joint homeostasis directly, through load-dependent matrix turnover, and indirectly, by preserving balanced mechanical loading across other joint components.

Because of their clinical importance, much of the available evidence relates to the cruciate and collateral ligaments of the knee. Human anterior cruciate ligament (ACL)-derived fibroblasts exposed to cyclic stretch showed increased type I and type III collagen mRNA expression, an effect mediated partly through autocrine transforming growth factor-β1 signaling [47]. Human ACL and medial collateral ligament (MCL) fibroblasts subjected to mechanical stretch also demonstrated differential matrix metalloproteinase-2 activity according to the applied stress, indicating that ligament cells may alter their matrix-remodeling behavior under injury-like mechanical conditions [48]. In rabbits, knee immobilization inhibited normal biomechanical maturation of the MCL and degraded its structural properties [49]. Conversely, appropriately dosed loading after ligament injury may improve tissue strength and stiffness compared with immobilization, although excessive or poorly timed loading may compromise healing quality [50].

ACL and MCL injuries alter joint kinematics and load distribution, with secondary effects on cartilage, menisci, synovium, and subchondral bone [51]. The strongest clinical inference is therefore biomechanical: ligament integrity preserves physiological movement patterns, whereas ligament insufficiency produces pathological misloading that may promote joint degeneration. Current evidence supports ligaments as movement-responsive tissues that are essential to joint mechanical homeostasis. Their direct contribution to synovial fluid composition remains insufficiently characterized, but their indirect contribution to joint-organ homeostasis is achieved through stabilization, load distribution, and prevention of pathological movement patterns.

The menisci extend the concept of movement-responsive joint homeostasis by acting as fibrocartilaginous regulators of load distribution, fluid pressurization, joint congruity, and mechanical stability. Meniscal fibrochondrocytes are exposed to regionally heterogeneous compression, tension, shear, fluid pressure, and fluid flow. In response, they modify extracellular matrix synthesis, catabolic enzyme expression, inflammatory signaling, and mechanotransduction pathways. Menisci therefore contribute to joint homeostasis both mechanically, by distributing loads and protecting articular cartilage, and biologically, through load-sensitive matrix turnover and inflammatory modulation.

Meniscal fibrochondrocytes also respond to mechanical loading through inflammatory and osteoclast-related mediators. Human meniscal fibrochondrocytes exposed to dynamic stimulation modified the expression of receptor activator of nuclear factor-κB (RANK) and its ligand RANKL, demonstrating a load-sensitive immunoregulatory and remodeling response [52]. In humans, loaded magnetic resonance imaging and running studies have shown that the menisci deform dynamically under both physiological and experimentally imposed loading conditions [53,54]. Meniscal tears, extrusion, and root tears are associated with altered load distribution, cartilage degeneration, incident or progressive osteoarthritis, and more rapidly progressive osteoarthritis phenotypes [55,56].

The glenoid labrum provides a comparable fibrocartilaginous example in the shoulder, where its principal contribution is mechanical stabilization through concavity-compression and control of humeral head translation. Histological and biomechanical studies demonstrate organized collagen architecture and regional specialization adapted to direction-specific loading [57,58]. However, unlike the knee meniscus, direct evidence of cellular mechanobiology in the labrum remains limited.

Menisci and labra therefore extend the movement-responsive joint concept beyond cartilage and synovium. Menisci are supported by substantial mechanical and mechanobiological evidence regarding their roles in load distribution, fluid pressurization, matrix turnover, and cartilage protection. The glenoid labrum contributes predominantly by stabilizing glenohumeral motion and preserving concavity-compression mechanics, although direct evidence of cellular mechanohomeostasis within the labrum remains limited.

Intra-articular adipose tissues, particularly the infrapatellar fat pad (IFP) of the knee, add a soft-tissue and immunometabolic dimension to movement-responsive joint homeostasis. Their anatomical location exposes them to compression, shear, deformation, and fluid displacement during movement, while their cellular composition enables responses through adipokine and cytokine release, stromal and vascular adaptation, immune regulation, and fibrotic remodeling [59]. Current evidence supports a dual role. Under physiological conditions, articular fat pads may contribute to cushioning, synovial fluid distribution, proprioception, and local trophic support. In contrast, abnormal loading, obesity-related inflammation, injury, or osteoarthritis may shift these tissues toward fibrosis, inflammatory mediator release, altered mechanics, and pain sensitization.

Human osteoarthritic IFP explants secrete adipokines and inflammatory mediators and can influence cartilage and synovial cells through factors released into conditioned media. The IFP has been shown to express higher concentrations of several inflammatory mediators than subcutaneous adipose tissue, including IL-6, adipsin, adiponectin, and visfatin [60]. Nevertheless, conditioned medium derived from end-stage osteoarthritic IFP inhibited several catabolic mediators in bovine cartilage explants, suggesting that its biological effects are context dependent rather than uniformly deleterious [61].

Voluntary wheel running in mice induced dynamic alterations in IFP cellularity and gene expression, synovial fluid metabolomics, collagen content, adipocyte size, myeloid-cell populations, oxidized phospholipids, and inflammatory mediator profiles. The initial inflammatory response was transient and resolved within the first days of exercise, whereas sustained daily activity was associated with a more homeostatic IFP response [62]. Pathological remodeling of the IFP in diet-induced obesity has also been shown to precede detectable osteoarthritic changes in the knee, supporting the possibility that this tissue can act as an early local amplifier of disease [63]. Specimens obtained from patients with end-stage osteoarthritis were found to be stiffer than several other adipose tissues, consistent with fibrosis impairing the normal force-distribution function of the tissue during movement [64].

Direct evidence that mechanical loading regulates articular fat-pad homeostasis in humans remains limited. Nevertheless, ex vivo biomechanical studies, animal exercise experiments, and analyses of clinical osteoarthritis specimens support its characterization as a deformable, innervated, and immunometabolically active tissue whose physiological or pathological remodeling may influence joint mechanics, inflammation, lubrication, and pain.

Joint sensation is itself mechanosensitive. Afferent fibers located within the capsule, synovium, ligaments, menisci, periosteum, and subchondral bone detect stretch, pressure, shear, and inflammatory changes, translating local mechanical conditions into proprioceptive information, protective reflexes, or pain [65]. Physiological movement may therefore contribute to sensory calibration, whereas inflammation, injury, overload, or prolonged abnormal loading may sensitize articular nociceptors and transform movement from a regulatory stimulus into a painful input.

Articular nociceptors in mice express mechanosensitive ion channels that convert mechanical forces into electrical activity. In experimental osteoarthritis, sensitized mechanosensitive ion channels contribute to mechanical allodynia, and intra-articular inhibition with GsMTx4 reduces dorsal horn activation and mechanical pain behavior [66]. Inflammatory cytokines and nerve growth factor (NGF) can also recruit previously mechanically insensitive afferents [67], potentially increasing sensitivity to mechanical loading. Animal electrophysiological studies further support the existence of neural afferents responsive to movement. Group III and IV articular afferents within the medial articular nerve are activated by mechanical stimulation and passive movement of the knee [68]. Piezo2-expressing nociceptors have also been shown to mediate mechanical sensitization in osteoarthritis-related pain and inflammation, linking a defined mechanotransduction pathway to joint pain behavior [69].

In patients with osteoarthritis, exercise-induced hypoalgesia is variable. Dysfunctional conditioned pain modulation is associated with an impaired exercise-induced hypoalgesic response, suggesting that movement-related pain relief depends partly on the integrity of descending inhibitory control [70]. A single exercise session reduced pain during and after exercise in individuals with knee osteoarthritis, whereas education regarding exercise-induced hypoalgesia did not significantly modify this response, suggesting that the observed effect was not explained solely by expectation or placebo mechanisms [71]. In a randomized controlled trial, mild-to-moderate pain experienced during knee exercise was associated with greater odds of an exercise-induced hypoalgesic response in individuals with knee osteoarthritis. This finding reinforces the concept that tolerable movement can engage pain-modulatory systems rather than necessarily aggravating symptoms [72]. In another randomized controlled trial, a supervised 12-week exercise program reduced pressure-pain sensitivity, temporal summation, and self-reported pain in patients with knee osteoarthritis compared with no exercise [73]. Dedicated interventions such as proprioceptive training have likewise been associated with improvements in osteoarthritis symptoms, quality of life, pain, balance, and joint-position sense, supporting the hypothesis that movement-based interventions can improve the sensorimotor dimension of joint function [74].

**Figure 4:**
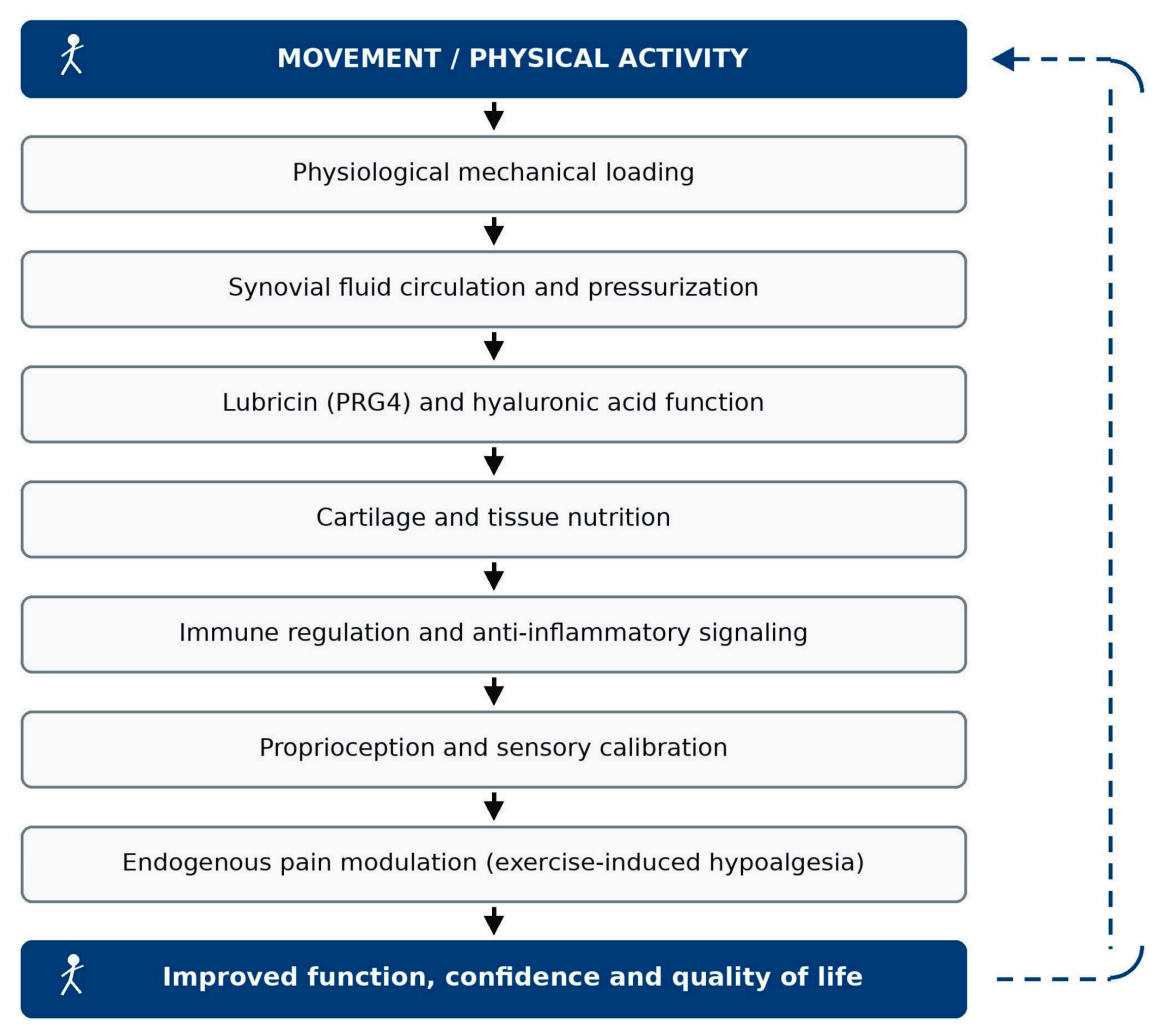
Movement Regulates Joint Biology and Pain.

Collectively, experimental and clinical evidence supports a role for movement in joint sensory regulation, but the response is heterogeneous. Mechanosensitive nociceptors provide a biological basis for load-related pain, whereas clinical studies demonstrate that exercise can engage hypoalgesic and proprioceptive mechanisms [65,66,67,68,69,70,71,72,73,74]. However, exercise-induced hypoalgesia is not universal and appears to depend on factors including descending pain inhibition, inflammation, central sensitization, and individual tolerance. Movement should therefore be considered a potential modulator of joint sensitivity rather than an inherently analgesic stimulus (Figure 4).

Considerable heterogeneity exists among experimental and clinical studies with respect to training modality, loading intensity, periodicity, duration, and exercise type. Individual responses are also likely to be influenced by previous training status, age, metabolic profile, body composition, inflammatory background, and associated comorbidities. Consequently, broad generalization remains difficult, and multidimensional relationships must be better defined before individualized movement-based protocols can be established for joint-pain modulation and functional recovery. Such protocols should aim not only to restore movement capacity, but also to rebuild confidence in movement and reduce joint sensitivity within an individualized adaptive loading range (Figure 4).

## 6. Inactivity as Amplifier of Pain and Dysfunction

Synovial joint tissues retain a lifelong mechanoresponsive phenotype that originates in developmental mechanosensitivity. Within physiological limits, mechanical loading supports tissue turnover, lubrication, structural adaptation, and the balance between inflammatory and regenerative processes. Conversely, overload, maldistributed stress, unloading, and inactivity can progressively impair homeostatic maintenance, promoting abnormal matrix remodeling, synovial inflammation, and loss of functional joint properties.

Inactivity, unloading, and immobilization deprive the joint of the cyclical mechanical signals required for synovial fluid turnover, cartilage nutrition, matrix remodeling, and sensorimotor calibration. In the absence of movement, the biological and mechanical conditions that preserve lubrication, tissue homeostasis, immune balance, and pain regulation are progressively disrupted.

In animal models, immobilization reduces synovial fluid hyaluronan concentration in parallel with changes in synovial intimal cell populations. In immobilized sheep joints, HA production, non-specific esterase (NSE) activity, and uridine diphosphoglucose dehydrogenase (UDPGD) metabolism in synovial tissue decrease. Both FLS and macrophage activity resume after remobilization, indicating that disuse can reversibly suppress joint metabolic activity [75]. In mice, combined knee immobilization and unloading induces early synovitis and fibrosis before cartilage degeneration, with single-cell analysis identifying activated fibroblast and macrophage subsets involved in inflammatory signaling [36]. Mechanical deprivation may therefore initially disturb synovial homeostasis and subsequently contribute to cartilage degeneration and joint contracture.

Cartilage thinning, altered cartilage matrix composition, and subchondral bone atrophy are established consequences of unloading, although their temporal relationship with synovial responses remains insufficiently characterized. In clinical settings, prolonged immobilization and bed rest rapidly reduce muscle mass, strength, bone mineral density, and functional capacity, creating the peripheral conditions for instability, altered gait, fear of movement, and further joint misloading [76,77]. Prolonged inactivity also modifies cartilage-related biomarkers, further supporting the mechanosensitivity of joint metabolism during disuse. Voluntary bed rest in healthy individuals increased biomarkers of cartilage catabolic activity, including COMP, MMP-3, MMP-9, and resistin, with only partial recovery after re-ambulation [78]. In a previous study, cartilage catabolic responses induced by five days of immobilization were not fully counteracted by simple re-ambulation or locomotion replacement training [79]. Evidence regarding the effects of disuse on other joint tissues remains more limited; however, the available data suggest that even in healthy individuals, the biological consequences of immobilization may not be fully reversed by resuming the same level of activity or by non-ambulatory mobilization alone.

Excessive, repetitive, or maldistributed mechanical loading can shift the joint from adaptive mechanotransduction toward tissue injury. At cellular and tissue levels, overload promotes chondrocyte catabolic signaling, extracellular matrix degradation, inflammatory mediator release, and cell death. In vitro, excessive mechanical loading of chondrocytes and cartilage induces gremlin-1, which activates NF-κB signaling and catabolic enzyme expression, thereby providing a possible link between overload and osteoarthritis-like cartilage degradation pathways [80]. Mechanical overload also increases mitochondrial superoxide production in chondrocytes while reducing superoxide dismutase 2 (SOD2) protein expression, supporting the existence of a mechanometabolic pathway of cartilage injury [81].

In bovine cartilage explants, both inflammatory cytokine exposure and mechanical injury reduced collagen synthesis, increased aggrecan expression, and altered matrix metalloproteinase profiles. Proteomic analyses identified responses related not only to tissue damage and inflammation, but also to growth and repair [82]. In an in vivo tibial cyclic compression model, controlled loading induced cartilage degeneration and subchondral bone changes depending on load magnitude, loading duration, and animal age, with distinct effects on metaphyseal and epiphyseal bone and on osteophyte formation [83]. Both instability and excessive repetitive loading can induce osteoarthritis-like pathology in ACL-deficient rat knees, with reduced subchondral bone in the injured joint and subchondral sclerosis in the contralateral knee [84]. ACL injury may also induce meniscal hypertrophy and mineralization, thereby contributing to cartilage degeneration and osteoarthritis progression.

Chronic abnormal cartilage loading induced by destabilization of the medial meniscus in rabbits produces early histological alterations in vivo, further supporting the relationship between focal misloading and cartilage damage [85]. Knee destabilization procedures are also widely used to induce post-traumatic osteoarthritis-like changes in rodent models, with disease progression occurring over species- and model-dependent timeframes. Although these models have traditionally focused on cartilage and subchondral bone, more recent evidence indicates that mechanical destabilization also profoundly affects synovial homeostasis. Early synovitis, activation of fibroblast and macrophage subsets, inflammatory mediator production, and fibrotic remodeling suggest that altered mechanics rapidly propagate to the synovial compartment and may precede or contribute to cartilage degeneration in both experimental models and clinical observational studies [86,87,88].

Taken together, the available evidence supports a nonlinear relationship between mechanical exposure and joint homeostasis. Both prolonged mechanical deprivation and excessive or maldistributed loading can promote adverse structural, inflammatory, and metabolic responses, although much of the mechanistic evidence derives from experimental models. The clinically relevant objective is therefore not maximal loading or maximal protection, but restoration of a tolerable mechanical range that supports adaptation without sustained tissue or symptom aggravation.

## 7. Movement as Pain Containment in Trauma, Osteoarthritis, and Healthy Aging

**Figure 5:**
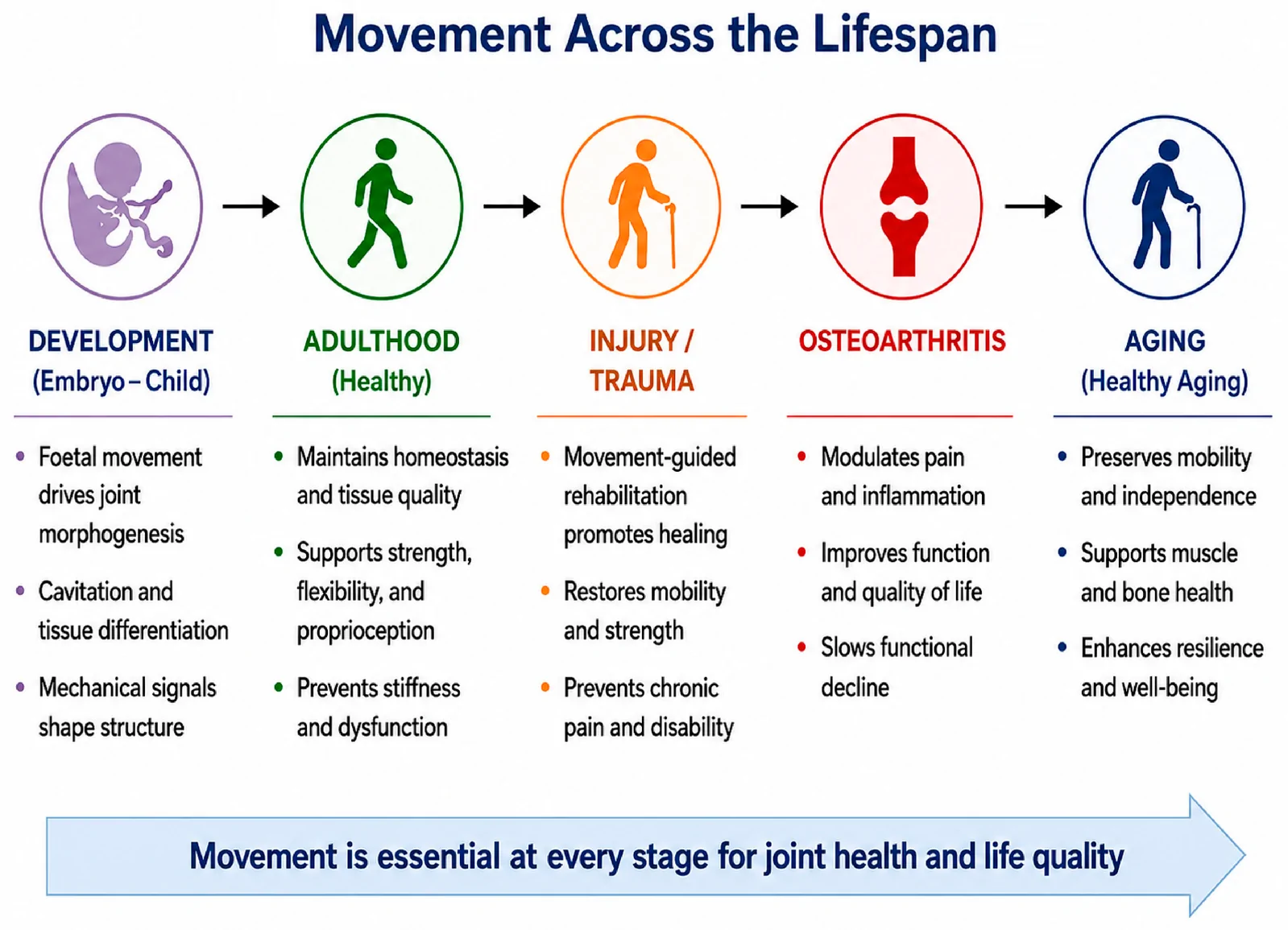
Movement Across the Lifespan.

Following joint trauma, particularly when articular surfaces are involved in load-bearing joints, prolonged immobilization or unloading has often been prescribed to protect cartilage and facilitate repair [89], although the biological evidence supporting extended mechanical deprivation as a regenerative strategy remains limited [90]. Temporary protection is necessary after acute injury or surgical repair; however, prolonged absence of movement may itself become a biological stressor by impairing synovial fluid turnover, cartilage metabolism, muscle activation, and sensory calibration (Figure 5).

Resuming movement after trauma should not be regarded merely as the restoration of mobility or physical performance, but also as the re-establishment of physiological mechanical and sensory signaling. Controlled loading after injury may help preserve lubrication, matrix turnover, tissue remodeling, and sensorimotor integration. In animal models, controlled postoperative or post-injury motion has often produced better cartilage repair or ligament mechanical properties than prolonged immobilization, whereas delayed, insufficient, excessive, or poorly timed loading may fail to reverse immobilization-induced damage or may impair healing quality [90,91,92].

In an ovine osteochondral scaffold model, partial postoperative loading improved mobility and scaffold integration compared with either immobilization or unrestricted full loading [93]. Similarly, rabbit osteochondral defect studies demonstrated that early continuous passive motion enhanced cartilage matrix repair and lubricin expression [94]. These findings support the concept that movement after trauma is beneficial when it is controlled and biologically tolerated, whereas both prolonged immobilization and excessive loading may compromise repair.

In clinical practice, rehabilitation after ACL reconstruction, osteosynthesis, and meniscal repair has progressively shifted toward earlier controlled mobilization when fixation or repair stability permits. Early-mobilization protocols may preserve functional recovery without clearly increasing laxity, fixation failure, or complication rates in appropriately selected patients, although the benefits vary according to tissue type, surgical procedure, and loading restrictions. A randomized controlled study comparing accelerated and non-accelerated rehabilitation after ACL reconstruction found no meaningful differences in knee laxity, clinical outcomes, functional performance, patient satisfaction, or synovial fluid biomarkers of cartilage metabolism during follow-up [95]. Unprotected weight bearing and mobilization as tolerated after osteosynthesis of Lauge-Hansen supination-external rotation type 2–4 ankle fractures improved functional outcomes and enabled earlier return to work and sports without increasing complications [96].

Immediate mobilization and supervised physiotherapy after volar locking plate fixation of unstable distal radius fractures resulted in significantly improved range of motion and grip strength at one year compared with postoperative casting [97]. Early weight-bearing rehabilitation initiated two weeks after syndesmotic ankle fracture fixation was also associated with earlier functional recovery, reduced pain, and faster return to work and normal daily activities [98].

Restoration of movement after trauma should therefore be understood as a controlled biological and functional process. Early movement may contribute to pain containment and recovery when repair stability, tissue biology, and individual tolerance are respected. Nevertheless, the transition from protection to loading does not follow a universal threshold. The appropriate timing and magnitude of movement depend on injury characteristics, the tissue involved, age, systemic conditions, previous activity level, the biological state of the joint, and a broad range of individual factors, including comorbidities, motivation, central pain processing, and sensorimotor integration. In this context, the challenge is not simply to determine when movement should resume, but to identify when the injured joint can once again interpret mechanical stimulation as a restorative rather than damaging signal.

Osteoarthritis provides one of the clearest examples of the dynamic interaction among movement, joint biology, and sensory perception. Traditionally, physical activity in osteoarthritis has been discussed mainly in relation to symptom reduction, muscle strengthening, weight management, and preservation of physical performance. However, movement may influence not only structural outcomes, but also the biological and perceptual conditions through which the joint is experienced in daily life.

From a primary prevention perspective, regular physiological activity may help maintain synovial fluid turnover, periarticular muscle support, proprioceptive competence, metabolic balance, and inflammatory equilibrium, thereby potentially delaying the development of maladaptive joint responses. Secondary prevention becomes relevant when early synovial activation, metabolic abnormalities, meniscal pathology, or initial symptoms emerge before extensive structural deterioration, corresponding broadly to Kellgren–Lawrence stages I and II. At this stage, movement may contribute to limiting the amplification of inflammatory signaling and sensory sensitization while preserving functional adaptation and confidence in movement.

In established disease, with radiographic changes corresponding to Kellgren–Lawrence stages III and IV, tertiary prevention becomes more relevant. At this stage, physical activity may no longer primarily target structural preservation, but instead focus on pain containment, maintenance of function, reduction of disability, and preservation of movement-related self-efficacy. This distinction is important because radiographic severity and symptom intensity frequently correlate only incompletely. Movement may influence exercise-induced hypoalgesia, proprioception, inflammatory balance, and central pain modulation even when structural disease is already present. From this perspective, physical activity is not merely an intervention applied to diseased tissue, but a continuous regulator of the biological and perceptual conditions that determine whether a joint feels painful, fragile, or functionally reliable.

Healthy aging reflects more than the preservation of musculoskeletal tissues; it also depends on maintaining integrated physiological responsiveness and the capacity to adapt to environmental demands. Movement contributes not only to preserving muscle mass and bone integrity, but also to maintaining proprioceptive competence, inflammatory balance, metabolic flexibility, and confidence in bodily function. Age-related changes progressively affect cartilage composition, subchondral bone architecture, muscle performance, sensory processing, and regenerative capacity, while systemic processes such as inflammaging and metabolic dysfunction further influence joint homeostasis. At the same time, aging is commonly accompanied by declining physical activity and increasing fear of movement, which may create a self-reinforcing cycle of inactivity, functional deterioration, and heightened pain sensitivity.

Within this context, movement acts not only through the preservation of structural integrity, but also by maintaining adaptive physiological responses. Recurrent physiological activity exposes tissues to appropriate mechanical stimuli, supports muscle–joint interactions, contributes to sensorimotor calibration, and may counteract biological processes associated with functional decline. Mobility is therefore relevant to healthy aging not only because it preserves physical performance, but also because it supports independence, reduces fall risk, and maintains engagement with the environment. Healthy longevity may consequently depend not merely on joints that remain anatomically intact, but on joints that continue to feel stable, usable, and reliable throughout life.

Movement may also influence pain-related behavior in older adults. Persistent pain can promote activity avoidance, reduced confidence, and progressive functional decline. Repeated tolerable activity may counter these processes by improving functional confidence and reducing movement-related fear, although individual responses remain influenced by pain severity, comorbidity, and sensory processing.

Across joint trauma, osteoarthritis, and healthy aging, the effects of movement appear to depend on biological context, loading magnitude, timing, and individual tolerance. These clinical settings can therefore be interpreted within the adaptive loading framework: the relevant loading range is dynamic and may shift with injury, disease severity, aging, inflammation, and recovery status.

## 8. Limitations

This review has several limitations. First, it was designed as a narrative and hypothesis-generating review rather than a systematic review; consequently, no formal risk-of-bias assessment, certainty-of-evidence grading, quantitative meta-analysis, or PRISMA-style record-level selection process was performed. Although a structured literature search was used, study selection necessarily involved author judgment and may therefore be subject to selection bias. Second, the evidence base is heterogeneous and includes human clinical studies, observational data, animal models, tissue explants, and cell-culture experiments. Mechanistic findings derived from experimental models cannot always be translated directly to human joint physiology or clinical rehabilitation. Third, substantial variation exists across studies in loading modality, intensity, duration, tissue studied, disease state, and outcome measures, limiting direct comparison and preventing definition of a universal dose–response relationship. Finally, the adaptive loading window proposed in this review is a conceptual and hypothesis-generating framework rather than a clinically validated threshold or predictive model. Its biological components, measurable boundaries, and clinical utility require prospective validation.

## 9. Conclusions and Future Directions

A central unresolved question emerging from the available evidence concerns the existence and determination of an adaptive loading window. Synovial joints do not appear to respond to movement according to a simple linear model in which increasing activity necessarily produces greater benefit. Instead, both insufficient and excessive mechanical stimulation may progressively disturb joint homeostasis. The position and breadth of this adaptive window are likely dynamic rather than fixed and may vary according to joint condition, previous injury, skeletal maturity, aging, sex, metabolic profile, inflammatory status, body composition, associated comorbidities, and physiological states such as development, pregnancy, or menopause. Previous training history and habitual movement patterns may also influence how mechanical stimuli are interpreted by the joint organ. The principal challenge may therefore not be to define a universal threshold separating beneficial from harmful activity, but to understand how an individual joint responds to a specific mechanical input at a particular biological moment.

A further distinction should be made between physiological movement and cumulative mechanical exposure. Not all physical activity necessarily generates adaptive responses. Occupational tasks characterized by prolonged standing, repetitive movements, constrained postures, asymmetric loading, or insufficient recovery may expose joints to sustained or maldistributed stress without reproducing the variability, coordination, and progressive adaptation associated with structured physical activity. Movement may also serve different objectives, ranging from athletic performance and physical fitness to preservation of self-care capacity, functional independence, and autonomy during aging or chronic disease. It should therefore be evaluated not only according to quantity or time spent active, but also in relation to variability, intensity, load distribution, recovery, biological tolerance, and intended functional purpose. A person who stands for prolonged periods at work, for example, may accumulate substantial mechanical exposure while remaining functionally underexposed to the forms of movement that support lubrication, sensorimotor integration, and adaptive tissue responses.

**Figure 6:**
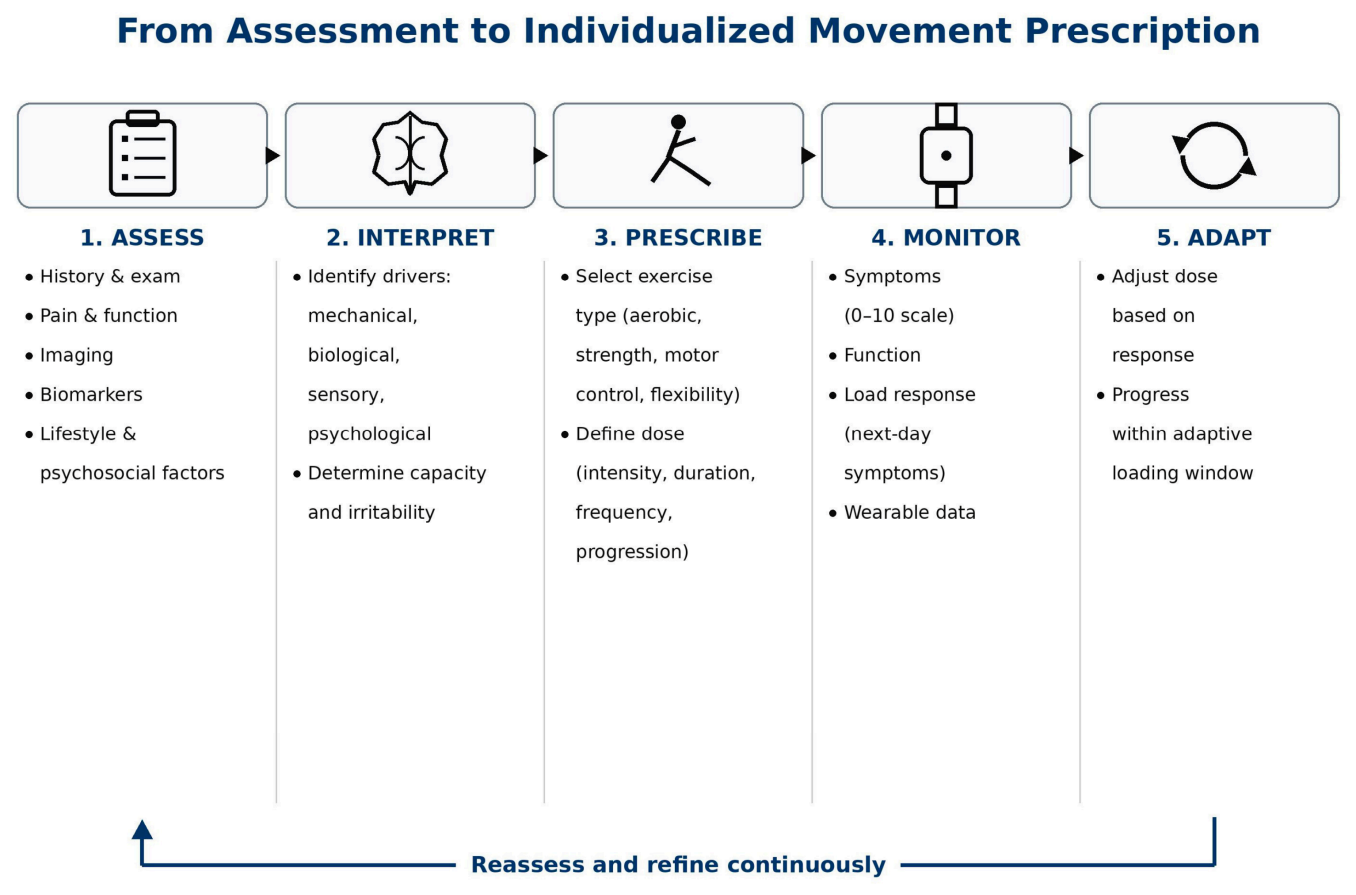
From Assessment to Individualized Movement Prescription.

From a clinical perspective, physiotherapists and rehabilitation specialists already estimate the balance between insufficient and excessive loading through symptoms, functional testing, strength, range of motion, gait analysis, fatigue, and tolerance to progression. However, current practice continues to rely largely on surrogate indicators, whereas the biological correlates that define an individual mechanobiological adaptive range remain insufficiently characterized (Figure 6).

The individualized adaptive loading window is unlikely to be identified through a single biomarker or functional test. More plausibly, it will require integration of biological markers of inflammation and matrix turnover, biomechanical assessments of loading and gait, wearable-derived measures of activity and recovery, patient-reported pain and confidence scores, and contextual variables such as sleep, fatigue, weather sensitivity, metabolic status, and comorbidities. Because joint sensitivity may fluctuate from day to day, the relevant clinical question may shift from “How much activity is generally safe?” to “What type and amount of movement can this joint tolerate, in this individual, under today’s biological and environmental conditions?”.

Movement may be considered tolerable not only when pain remains acceptable during activity, but also when subsequent symptoms, swelling, and stiffness remain limited and return to baseline within an appropriate recovery interval. The response of the joint on the following day may therefore represent one of the simplest clinically accessible indicators of whether the preceding activity remained within the adaptive window or exceeded it. Environmental modifiers, including temperature, humidity, and barometric pressure, may further influence symptom perception in susceptible individuals, reinforcing the importance of repeated rather than single-point assessment.

Future individualized movement prescriptions will therefore likely depend on an integrated profile rather than on a single threshold. Biological markers, imaging evidence of synovitis or bone marrow involvement, wearable-derived movement patterns, functional tests, and patient-reported measures of pain, stiffness, confidence, and recovery time could collectively define a personal adaptive loading profile. This profile would necessarily remain dynamic, as joint sensitivity may vary according to systemic inflammation, fatigue, sleep, metabolic status, hormonal state, weather conditions, and previous-day loading. The clinically relevant target is therefore not merely the structurally tolerable load, but the range of movement within which the joint remains functional, subjectively reliable, and free from progressive sensitization. From this perspective, future movement-based care may increasingly depend on identifying and adapting the form, dose, and timing of movement that best support joint homeostasis, pain regulation, and sustained function.

## Data Availability

No new data were created or analyzed in this study. Data sharing is not applicable to this article.

## Abbreviations

ACL	Anterior cruciate ligament
ATP	Adenosine triphosphate
COMP	Cartilage oligomeric matrix protein
COX-2	Cyclooxygenase-2
CREB	Cyclic AMP response element-binding protein
DMM	Destabilization of the medial meniscus
EIH	Exercise-induced hypoalgesia
FLS	Fibroblast-like synoviocytes
HA	Hyaluronan
HAS3	Hyaluronan synthase 3
IFP	Infrapatellar fat pad
IL-6	Interleukin 6
KL	Kellgren–Lawrence
LRT	Locomotion replacement training
MCL	Medial collateral ligament
MMP	Matrix metalloproteinase
MMS	Minimized stress model
MRI	Magnetic resonance imaging
NF-κB	Nuclear factor kappa B
NGF	Nerve growth factor
NSE	Non-specific esterase
OA	Osteoarthritis
PGE2	Prostaglandin E2
PRG4	Proteoglycan 4
PTHrP	Parathyroid hormone-related protein
RANK	Receptor activator of nuclear factor-κB
RANKL	Receptor activator of nuclear factor-κB ligand
scRNA-seq	Single-cell RNA sequencing
SF	Synovial fluid
SOD2	Superoxide dismutase 2
TGF-β1	Transforming growth factor beta 1
UDPGD	Uridine diphosphoglucose dehydrogenase
YAP	Yes-associated protein
